# Ahead, a Novel Validated Machine Learning Model for Clinical Diagnosis of Alcohol Associated Hepatitis

**DOI:** 10.21203/rs.3.rs-8960896/v1

**Published:** 2026-05-26

**Authors:** David Malicote, Gene Im, Ethan Weinberg, Allison Kwong, SM Shasthry, Archana Rastogi, Rashmi Tondon, Nipun Verma, Arun Valsan, Paul Kwo, Rajender Reddy, Shiv Sarin, Winston Dunn, Ashwani K. Singal

**Affiliations:** University of Louisville School of Medicine; Mount Sinai Medical Center; University of Pennsylvania; Stanford University; Institute of Liver and Biliary Sciences; University of Pennsylvania; University of Pennsylvania; Post Graduate Institute of Medical Education and Research; Amrita Institute of Medical Sciences and Research Centre; Stanford University; University of Pennsylvania; Institute of Liver and Biliary Sciences; University of Kansas Medical Center; University of Louisville School of Medicine

**Keywords:** AI, Cirrhosis, AH, Biopsy

## Abstract

**Background:**

Current criteria from the National Institute on Alcohol Abuse and Alcoholism (NIAAA) for clinical diagnosis are not based on evidence. We developed a machine-learning model for the clinical diagnosis of AH.

**Methods and Results:**

467 subjects with alcohol associated liver disease (ALD) across seven centers were randomly assigned to training or testing cohorts. Three machine learning algorithms: Random Forest, Gradient Boosting Machine, and XGBoost were derived on the training cohort to identify clinical characteristics associated with histologic AH. Combined Alcohol Hepatitis Ensemble Algorithm Development (AHEAD) model integrating AST, ALT, total serum bilirubin was superior to NIAAA criteria to predict histologic AH in test cohort AUC of 0.695 (0.608–0.782) vs. 0.590 (95% CI 0.49420100330.685) (Bootstrap p = 0.049). Using 50% probability cutoff, AHEAD model was 61.5% specific (107 of 174 non-cases correctly identified) and 63.3% sensitive (19 of 30 cases correctly identified) for AH diagnosis. NIAAA model was 54.6% specific (95 of 174 non-cases correctly identified) and 63.3% sensitive (19 of 30 cases correctly identified).

**Conclusion:**

The AHEAD model (https://aihepatology.shinyapps.io/AHEAD/) is more specific than the widely utilized NIAAA criteria for clinical diagnosis of AH. It may be useful in clinical trials by reducing enrolment of subjects without AH.

## INTRODUCTION

Alcohol-associated hepatitis (AH) is a clinical syndrome characterized by new or worsening jaundice in the setting of heavy alcohol use. In severe form (MELD > 20), AH is often associated with multi-organ failure and high mortality.^1^ Corticosteroids, the only available pharmacotherapy for a severe episode of AH, is imperfect with several limitations including ineligibility to steroids in about 50% cases, response rate of 50–60% in those who are eligible, ability to identify responders only after using steroids for 4–7 days, and risk of bacterial and fungal infection, especially in non-responders. Given the high mortality of AH and limited treatment options, there are several ongoing clinical trials for additional therapies that could improve outcomes for this population.^2^ Additionally, there have been increased efforts to offer an early liver transplant to patients presenting with severe AH, however only 2–3% with severe AH are candidates.^3^

It is important to make an accurate diagnosis of AH in a patient presenting with decompensated alcohol-associated liver disease (ALD), especially when considering corticosteroids or enrolling in clinical trials. The gold standard for diagnosis of AH is liver biopsy, defined by hepatocyte ballooning, lobular inflammation with neutrophils, Mallory hyaline, and pericellular fibrosis.^4^ As transcutaneous liver biopsy poses risks of procedural bleeding, a trans jugular liver biopsy is recommended, which may not be available at some centers as this requires specialized interventional radiology expertise.^5^ The National Institute on Alcohol Abuse and Alcoholism (NIAAA) AH Consortia recommended criteria for clinical diagnosis of AH, and reserve liver biopsy when clinical diagnosis is uncertain. These criteria include: AST > 50, AST and ALT < 400 IU/L with AST/ALT ratio ≥ 1.5, serum bilirubin > 3 mg/dL, last alcohol use within 60 days of presentation, and exclusion of other causes of liver disease and of acute presentation.^6^ These criteria are currently used for enrolment in clinical trials for AH, however, their sensitivity is 63% specificity is 78% for histologic diagnosis of AH.^7^ A newer diagnostic model called NIAAA-CRP using the NIAAA criteria (lower cut-off of serum bilirubin at 2.5 mg/dL)with CRP ≥ 10mg/L has been shown to improve specificity to 82%, with a similar sensitivity of 72%, indicating that subtle modification of the NIAAA criteria with additional clinical variables can improve diagnostic accuracy.^8^

Artificial intelligence (AI) is being utilized more frequently for the development and optimization of diagnostic and prognostic models for liver disease.^9^ Machine learning allows for analysis of complex medical data and identification of trends that can be helpful in diagnostics. Specific to AH, AI and machine learning has helped derive models to predict prognosis and mortality in severe AH better than MELD score, and predicting likelihood of relapse to alcohol use after liver transplant.^10,11^ Our primary objective of this study is to use AI and machine learning approach to develop a better model for clinical diagnosis of AH.

## RESULTS

### Study Population

**Figure 1:**
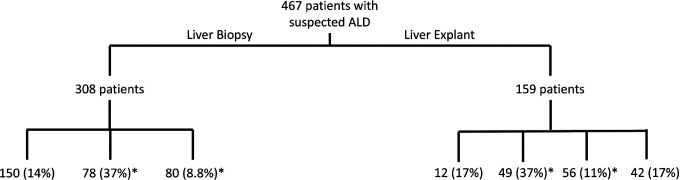
Randomization of contributing centers to the training and testing cohorts. The centers randomized to the training cohort are indicated by an asterisk. The numbers within parenthesis indicate percentage of subjects with histologic diagnosis of alcohol-associated hepatitis (AH).

Patients with clinical diagnosis of ALD and available liver histology data on a liver biopsy or explant were included in this analysis from seven hepatology centers. Of 467 patients, 263 from four centers were assigned randomly to the training and the remaining 203 patients from other three centers to the testing cohort (Fig. 1). The training cohort included 158 patients from two centers with liver histology from a biopsy (23% had AH on histology) and 105 patients from two centers with liver histology from an explant (23% had AH on histology). The testing cohort had 150 patients from one center with liver histology from a biopsy (14% had AH on histology) and 54 patients from two centers with liver histology from an explant (17% with AH on histology).

### Baseline Characteristics

The baseline demographic and clinical characteristics of patients included in the analysis were median (interquartile range or IQR) age of 45 (38–53) yrs., 89% males, median (IQR) MELD score of 31 (22–40). With a median of 42 (20–90) days to initial presentation from the last alcohol drink, a total of 283 (60.6%) patients met the NIAAA criteria for the clinical diagnosis of AH, and 90 (19.3%) had confirmed AH on liver histology (Table 1). The training as compared to the testing cohort had patients who were younger and had a higher MELD score. Further, with a numerically shorter median time to presentation since the last alcohol drink of 37 vs. 52 days (p = 0.237), patients in the training vs. testing cohort more often met the NIAAA criteria. Other baseline characteristics of the study population are illustrated in Table 1. The prevalence of AH on liver histology was also higher in the training vs. testing cohort (22.8 vs. 14.7%, P = 0.037). The two cohorts stratified for AH on histology (Table 2) were similar for most baseline characteristics except for days since last alcohol use. For example, patients with presence (N = 30) vs. absent (N = 174) AH on histology in the testing cohort had shorter median duration since last alcohol use (20 vs. 60 days, P = 0.004). In the training cohort, there was a trend for shorter median duration since the last alcohol use of 30 days in 60 patients with histologic AH as compared to 45 days in 203 patients without AH on histology, P = 0.060 (Table 2).

### Liver Histology Findings

**Figure 2:**
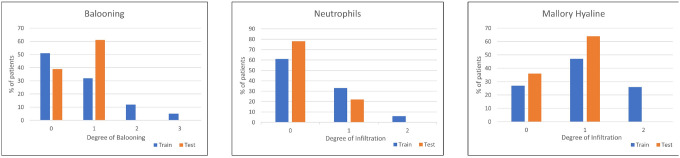
Percentage of subjects with hepatocyte ballooning, Mallory Hyaline, and neutrophilic infiltration on liver histology in the training and testing cohorts.

Figure 2 shows the individual histological characteristics of each cohort. Patients in the training cohort more often had neutrophilic lobular inflammation and Mallory’s hyaline, while testing cohort more often had hepatocyte ballooning (61 vs. 49%, P < 0.001). The severity of histological findings also varied across the two cohorts. For example, the training cohort had more severe histology for all components including hepatocyte ballooning (numerical score of 2 or 3), neutrophilic lobular inflammation (numerical score of 2), and Mallory hyaline (numerical score of 2) in 16%, 6%, and 26% respectively compared to none of the patients in the testing cohort for all the components. As expected, all the components on liver histology (hepatocyte ballooning, Mallory Denk bodies, and neutrophilic lobular inflammation) were observed more often in patients with vs. without AH on liver histology (**Supplementary Fig. 1**).

### Generation of Models in the Training Cohort

**Figure 3:**
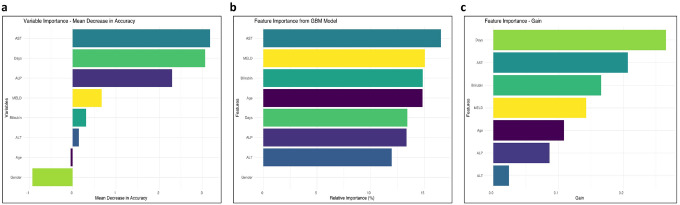
Variable order of importance as determined by the Random Forest (a), Gradient Boosting Machine (b), and XG Boost (c) prioritized by change in accuracy in the identification of AH in the training cohort. RF prioritized variables by mean change in accuracy, whereas GBM and XGB prioritized features by relative importance and gain, respectively.

Using the clinical variables from the training cohort, three machine learning models (RF, XGBoost, and GBM) were developed to predict histological AH as defined on the liver histology. Variable importance was determined for each model (Fig. 3a–c). RF determined variable importance to be greatest for AST followed by days since the last alcohol drink, serum alkaline phosphatase level, and MELD score. GBM determined that all the variables to be important in the model except for patient’s gender. XGB determined the greatest variable importance to be days since last drink followed by AST, bilirubin, and MELD score.

### Calibration of Predictive Models and Generation of Ensemble Model in Testing Cohort

**Figure 4:**
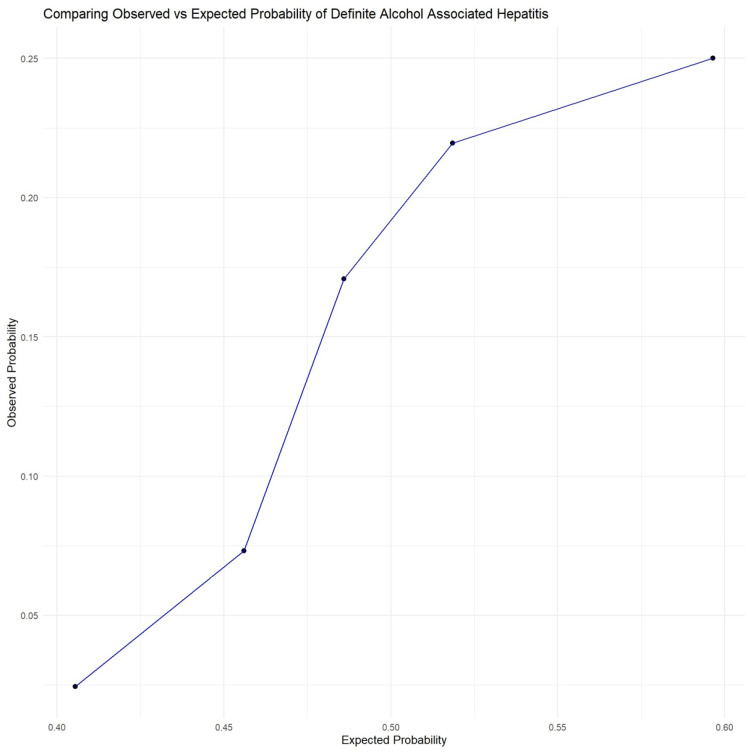
Calibration of the Alcohol-associated Hepatitis Ensemble Algorithmic Development (AHEAD) model on the observed vs. expected probability of alcohol associated hepatitis on liver histology (definite AH) in the testing cohort.

The three models developed in the training cohort were calibrated against the NIAAA diagnostic model for the clinical diagnosis of AH in the testing cohort. The models’ accuracies on the probability of predicting AH on histology as defined by Brier score were 0.329, 0.278, and 0.162 for RF, XGboost, and GBM models respectively. The respective areas under curve (AUCs) and 95% confidence intervals of the receiver operating characteristic (ROC) for these three models in the testing cohort were 0.682 (0.592–0.780), 0.697 (0.608–0.787), and 0.664 (0.571–0.757), respectively. The AH Ensemble Algorithm Development (AHEAD) model was developed using the geometric mean of the three machine learning models and was calibrated against the NIAAA diagnostic model in the testing cohort which revealed a Brier score of 0.259 and corresponding AUC (95% CI) of 0.693 (0.606–0.780). The calibration curve of the AHEAD model is shown in Fig. 4. The AUC for the NIAAA criteria for the clinical diagnosis of AH in the test cohort was 0.590 (0.481–0.699). Comparison of three machine learning models, AHEAD model, and NIAAA criteria for clinical diagnosis of AH in the testing cohort showed AHEAD model to be the best model (Fig. 5a).

### Comparison of the Ensemble model to the NIAAA model in the Testing Cohort

Figure 5b shows the ROC curve of the AHEAD model compared to that of the NIAAA criteria with a Bootstrap value of P = 0.049, indicating superior accuracy of the AHEAD model in the clinical diagnosis of AH. Using a 50% probability cutoff, the AHEAD model was 61.5% specific (107/174 non-cases) and 63.3% sensitive (19/30 cases). Using the same probability cutoff, the NIAAA model has the same sensitivity of 63.3%, but a lower specificity of 54.6% (95/174 non-cases).

## DISCUSSION

AH has high morbidity and mortality especially in severe disease (MELD > 20), and its treatment options are limited. While steroids improve short term outcomes for the first month in patients with severe AH,^13^ there is no medium and/or long-term benefit beyond this point.^14^ Further, steroids are prone to increase the risk of infection in these sick patients, especially for those who do not respond. Thus, accurate clinical diagnosis of AH is essential whenever corticosteroids are considered as a treatment option. NIAAA criteria are currently used for clinical diagnosis of AH are not based on evidence. In this study, we showed that the AHEAD model, which utilized a machine learning approach, is more specific as compared to the NIAAA criteria, while retaining the same sensitivity in making a clinical diagnosis of AH, with the gold standard being histologic AH. Thus, our new model is better to exclude patients who do not have AH on liver histology.

In our cohort, we found the NIAAA criteria to be less specific (55%) than what had been reported as 78% and 81% previously.^7,8^ This is in part due to higher median MELD of 22 in our study as compared to median MELD score of 16 and 17 in these respective studies.^7,8^ In a subgroup analysis on severe AH (MELD > 20) from one of these studies, the specificity of NIAAA criteria alone and when combined with C-reactive protein (CRP) levels decreased to 27% and 33% respectively. Inclusion of Mallory-Denk bodies as a criterion on liver histology for diagnosis of histological AH may also explain a lower specificity of the NIAAA criteria in our study compared to previous reports. In a meta-analysis, the inclusion of Mallory bodies as a histologic criterion for AH diagnosis reduced the precision of clinical diagnosis from 86% to 66%.^16^

The findings of our study have important implications in clinical practice and research. Using the AHEAD model for clinical diagnosis of AH will provide more precision in excluding cases who do not have AH on liver histology.^15^ Similarly, using the AHEAD model for clinical diagnosis will help with enrollment in clinical trials and reduce the risk of including patients who do not have AH. A link to the AHEAD model: https://aihepatology.shinyapps.io/AHEAD/. Using the same criteria for histological diagnosis of AH as in our study, with an accuracy of 59% (sensitivity 63% and specificity 55%) for NIAAA criteria, 70 additional patients for every 100 patients would be needed in the study to avoid misclassification of histological AH. In the same scenario, using the AHEAD model for clinical diagnosis with accuracy of 63% (sensitivity 63% and specificity 62%), only 59 patients for every 100 patients would be needed in the study to avoid misclassification of histological AH. Hence to recruit 300 AH patients mimicking histological AH for a study power of 80%, 510 patients would be needed using the NIAAA criteria and only 477 patients using the AHEAD model, avoiding misclassification and retaining the same study power.

Including patients from multiple centers and machine learning approach are strengths of our study, however, our study has limitations. For example, a retrospective study design has the potential of introducing selection bias along with having limitations of any retrospective study. A variable time gap in obtaining liver tissue for histology especially for explants is another limitation as it is known that histologic findings can fade and disappear with time. This makes it difficult to discern whether the model is capturing temporal bias or inherent features of AH. This combined with a set of rigorous criteria to define histological AH may have led to a lower prevalence of AH at 20% on liver histology in our cohort. Using the histological findings as reported in the medical charts rather than reassessment of histological slides by two pathologists with expertise on AH histology assessment may have contributed to the low prevalence and limit the generalizability of the study findings. As the CRP is not routinely tested in the management of AH patients, we were unable to examine comparison of our model against NIAAA-CRP model which has been shown to be better than NIAAA criteria.^8^ Further, predominantly male population accounting for 89% of the study cohort is another limitation, given the rapid and disproportionate rising incidence of AH in women in the US.^17^

In summary, we have developed a novel, validated machine-learning model (AHEAD) for the clinical diagnosis of AH. The superior specificity of our model vs. the NIAAA criteria would better avoid administering steroids to non-AH patients and reduce enrollment of non-AH patients in clinical trials, impacting study power. Studies are also needed to derive simple and uniform criteria on liver histology for diagnosis of AH.

## METHODS

### Study Design and Population

Seven centers with liver histology data (3 with liver biopsy data and 4 with liver explant data) for patients with ALD were included in the study. Study was approved at the local IRB at the University of Louisville IRB and at every center. The study qualified for consent waiver given retrospective chart review and data collection. The data from each site was shared as a deidentified file. The study was conducted in accordance with the Declaration of Helsinki with the experimental approach was approved by the institutional review board committee.

### Data Collection and Definitions

Investigators at each center performed a medical chart review to collect data on patients with ALD for pre-identified variables. Diagnosis of ALD was confirmed with heavy alcohol use (> 50 g/d for women or > 60 g/d for men) and exclusion of other causes of liver disease. Clinical data was extracted for patient demographics (age, sex, race), dates (last drink, biopsy, steroid start and end, death, last available follow up on survivors), baseline labs within one month of biopsy for biopsied cohorts or at the time of liver transplant listing for explants (AST and ALT in IU/liter, White blood count or WBC, platelets, serum sodium in mEq/liter, serum creatinine in mg/dL, serum bilirubin in mg/dL, INR, Prothrombin time of the patient and control). Histology data was extracted for steatosis, hepatocyte ballooning, neutrophilic infiltration, Mallory hyaline, ductular cholestasis, hepatocyte cholestasis, megamitochondria, fibrosis and stage. The degree of hepatocyte ballooning, Mallory-Denk bodies, and neutrophilic infiltration were graded on a scale of 0 to 2 (0 to 3 for hepatocyte ballooning) based on the SALVE histopathological staging protocol.^12^ The outcome of AH was defined by histologic presence of neutrophilic lobular inflammation, hepatocyte ballooning, and Mallory-Denk bodies.

### Statistical analysis

#### Center randomization

The seven centers were randomly assigned to either the training or testing cohort. Four centers (liver histology from biopsy and from explant in two centers each) were assigned to the training cohort. Three centers (liver histology from explant from two centers and biopsy from one center) consisting of 204 patients were assigned to the testing cohort.

#### Machine learning

From the training cohort, three machine learning algorithms- Random Forest (RF), Gradient Boosting Machine (GBM), and XGBoost (XGB)- were developed. These three algorithms were chosen due to their complementary strengths of handling diverse data, bias reduction, and ability to prevent overfitting. Each model underwent hyperparameter tuning using the training dataset. The models’ predictive performance was determined using out-of-bag cross-validation (OOB CV) to compute the area under the curve (AUC). The intrinsic structures of RF, GBM, and XGB algorithms account for all possible pairwise interactions, capturing complex relationships among variables. The final ensemble model, termed the Alcohol Hepatitis Ensemble Algorithm Development (AHEAD), was constructed as a geometric mean of the three models.

#### Validation and comparison

The ensemble AHEAD model was validated in the testing cohort. To assess its predictive value, the AUC and its 95% confidence interval (CI) were determined using DeLong’s method. AUC is a fundamental performance metric in machine learning to quantify the ability of the model to differentiate between positive and negative class across all potential classification thresholds. The ensemble model was then compared to the clinical diagnosis of AH using the NIAAA criteria. Differences in AUC values and the potential incremental predictive value of additional variables were evaluated using DeLong’s test and the Log-Likelihood Ratio test.

## Supplementary Material

Supplementary Files

This is a list of supplementary files associated with this preprint. Click to download.

- Supplementarydocument.docx

## Figures and Tables

**Figure 5:** Receiver operating characteristic (ROC) curve of the ensemble Alcohol-associated Hepatitis Ensemble Algorithmic Development (AHEAD) model vs. a) ROC curves of Random Forest (RF), Gradient Boosting Machine (GBM), XG Boost (XGB), and National Institute of Alcoholism and Alcohol Abuse (NIAAA) criteria and b) NIAAA criteria. The “Source” key in each panel indicates which line represents each model’s performance.

**Table 1:** Baseline characteristics of the training and testing cohorts

AH N (%)		Train Cohort (N = 263)	Test Cohort (N = 204)	Total (N = 467)	P
		**60 (22.8)**	**30 (14.7)**	**90 (19.3)**	**0.037**
Age	Median (IQR)	42.0 (36.0 to 49.0)	49 (42 to 56)	45 (38 to 53)	**<0.001**
Gender	Female N (%)	35 (13.3)	18 (8.8)	53 (11.3)	**0.171**
	Male N (%)	228 (86.7)	186 (91.2)	414 (88.7)	
AST IU /Liter	Median (IQR)	94.0 (66 to 153)	178 (122 to 260)	133 (74.5 to 219)	**<0.001**
ALT IU/Liter	Median (IQR)	42.0 (28.0 to 67)	90.5 (69 to 129)	61 (32 to 99)	**<0.001**
ALP IU/Liter	Median (IQR)	117.0 (89 to 166)	56.5 (36 to 93.2)	95 (57 to 141)	**<0.001**
MELD	Median (IQR)	37.0 (29 to 41)	22 (18.8 to 30)	31 (22 to 40)	**<0.001**
Bilirubin mg/dL	Median (IQR)	18.9 (9 to 30)	7.8 (5.2 to 14.6)	12.1 (6.5 to 25.1)	**<0.001**
Days to presentation since the last alcohol drink	Median (IQR)	37.0 (20 to67)	52 (14 to 105)	42 (19.5 to 90)	**0.237**
NIAAA criteria	No N (%)	78 (29.6)	106 (52)	184 (39.4)	**< 0.001**
	Yes N (%)	185 (70.4)	98 (48)	283 (60.6)	

**AH**: *Alcohol-associated hepatitis*; **IQR**: *Interquartile range; AST: Aspartate aminotransferase; ALT: Alanine aminotransferase; ALP: Alkaline phosphatase; MELD: Model for end-stage liver disease; NIAAA: National Institute of Alcoholism and Alcohol Abuse*.

**Table 2:** Baseline characteristics of the training and testing cohorts stratified by histologic presence or absence of AH.

		Train Cohort (N = 263)			Test Cohort (N = 204)		
		NoAH (N = 203)	AH (N = 60)	P	NoAH (N = 174)	AH (N = 30)	P
Age	Median (IQR)	42 (35 to 50)	41 (36 to 46.2)	0.404	49 (42 to 56)	46.0 (38 to 58)	0.336
Gender	Female (%)	26 (12.8)	9 (15)	0.824	14 (8)	4 (13.3)	0.552
	Male (%)	177 (87.2)	51 (85)		160 (92)	26 (86.7)	
AST	Median (IQR)	94.0 (66 to 156)	95 (69 to 140)	0.988	181 (125 to 262)	154 (114 to 228)	0.345
ALT	Median (IQR)	41 (28 to 66)	45 (27.2 to 71)	0.960	92 (71 to 138)	82.5 (57 to 106)	0.368
ALP	Median (IQR)	121 (88 to 171)	111 (91 to 157)	0.603	54.5 (36 to 90)	71 (50 to 104)	0.239
MELD	Median (IQR)	38 (30 to 42)	36 (25 to 40)	0.054	22 (18.2 to 30)	21.5 (19 to 30)	0.565
Bilirubin	Median (IQR)	16.9 (8.6 to 29)	22.8 (9.5 to 32.1)	0.078	7.8 (5.2 to 14.4)	7.9 (4.6 to 22.4)	0.808
Days	Median (IQR)	43 (24.5 to 74.5)	30 (16.8 to 60)	0.060	60 (18.5 to 108)	20 (4.0 to 54.5)	0.004
NIAAA Criteria	No (%)	59 (29.1)	19 (31.7)	0.820	95 (54.6)	11 (36.7)	0.106
	Yes (%)	144 (70.9)	41 (68.3)		79 (45.4)	19 (63.3)	

**AH**: *Alcohol-associated hepatitis*; **IQR**: *Interquartile range; AST: Aspartate aminotransferase; ALT: Alanine aminotransferase; ALP: Alkaline phosphatase; MELD: Model for end-stage liver disease; NIAAA: National Institute of Alcoholism and Alcohol Abuse*.

## Data Availability

The data sets generated during and/or analyzed during the current study are available from the corresponding author on reasonable request.

## Model Availability:

The model is available online at https://aihepatology.shinyapps.io/AHEAD/
